# The Potential Role of Increasing the Release of Mouse β- Defensin-14 in the Treatment of Osteomyelitis in Mice: A Primary Study

**DOI:** 10.1371/journal.pone.0086874

**Published:** 2014-01-28

**Authors:** Chen Zhu, Jiaxing Wang, Tao Cheng, Qingtian Li, Hao Shen, Hui Qin, Mengqi Cheng, Xianlong Zhang

**Affiliations:** 1 Department of Orthopaedic Surgery, Shanghai Sixth People’s Hospital, Shanghai Jiao Tong University School of Medicine, Shanghai, China; 2 Department of Orthopaedic Surgery, Anhui Provincial Hospital of Anhui Medical University, Hefei, China; 3 Department of Medical Microbiology and Parasitology, Shanghai Jiao Tong University School of Medicine, Shanghai, China; UNIFESP Federal University of São Paulo, Brazil

## Abstract

Mammalian β-defensins are small cationic peptides that have been implicated in mediating innate immune defenses against microbial infection. Mouse β-defensin-14 (MBD-14), based on structural and functional similarities, appears to be an ortholog of human β-defensin-3 (HBD-3). Previous studies identified signaling pathway p38 mitogen-activated protein kinase (MAPK) that contributed to the expression of MBD-14 in mouse osteoblasts upon contacted with methicillin-resistance *Staphylococcus aureus* (MRSA) supernatant, which provided a theoretical basis as a promising therapeutic target in the treatment of intramedullary infection with MRSA in vivo. In this study, the medullary cavities of tibiae were contaminated with MRSA 10^3^ colony forming units and different doses of p38 MAPK agonists anisomycin were followed as group III or IV in 30 mice. Fifteen animals that received phosphate- buffered saline served as group II and 15 mice were not contaminated with MRSA and received phosphate-buffered saline served as controls (group I). Follow-up was 7 days. In day 1, day 4 and day 7 postoperatively, infection was evaluated by blood routine, microbiological and histological analyses after sacrifice. All animals of group II developed microbiological and histological signs of infection. Histological signs of infection, white blood counts and cultures of group III and IV showed significantly reduced bacterial growth compared to cultures of group II. Simultaneously, different doses of anisomycin significantly induced the expression of osteoblast-associated genes, including alkaline phosphatase, osteocalcin and collagen type I. In addition, the expression of HBD-3 in human interfacial membranes around infected periprosthetic joint by *staphylococcus* contaminated was evaluated, and the expression pattern changed with significant induction of HBD-3 in infected periprosthetic joint compared with aseptic loosening under inflammatory conditions. Our primary study indicated that the potential antibacterial role of increased MBD-14 in the osteomyelitis mouse model.

## Introduction

Currently, the rate of infection following orthopaedics is low, but the treatment of osteomyelitis or bone infections are still difficult in clinical practice [1]. Considering the high number of orthopedic procedures performed annually, infection is not only a medical but also an economical problem and the consequences can be devastating, leading to prolonged hospitalization, poor functional outcome, and sepsis [2], [3]. *Staphylococcus aureus* (*S. aureus*), one of the major pathogens in implant-related or nosocomial infections, represents the most common bacterial cause of osteomyelitis and can be isolated in 80% of cases [4], [5]. Antimicrobial prophylaxis, including the systemic and local use of antibiotics, has proven to be valuable in the prevention of bone infection in clinical use [6]. Unfortunately, the misuse of antibiotics has led to the evolution and rapid spread of clinical bacterial strains resistant to the available antimicrobial agents [6], [7]. As a result, an increasing number of infections involving methicillin-resistant *S. aureus* (MRSA), even ‘Superbugs’, have emerged [4], [8]. Presently, the global spread of MRSA is a matter of great concern in the treatment of staphylococcal infection, since it has rapidly acquired resistance to many clinical antibacterial agents [9]. Thus, more effective antimicrobial agents for systemic or local prophylaxis or treatment against these antibiotic-resistant organisms must be investigated. In light of this situation, antimicrobial peptides are attractive candidates as therapeutic agents for bacterial infections because of their selectivity, speed of action, relative difficulty in production of resistant mutants, and inherent immunological compatibility [10].

A major family of antimicrobial proteins in mammals comprises the β-defensins. β-defensins are small (2 to 6 kDa), cationic proteins which exhibit a potent anti- microbial activity at microto nanomolar concentrations [11]. Previous studies have demonstrated that β-defensins were able to activate the innate immune system within the bone and may have a potential application for reducing the rate of peri-implant infections [12]. Human β-defensin-3 (HBD-3) is characterized by its strong, broad- spectrum, and salt-insensitive antibacterial activity against many bacteria, including multiresistant strains, such as MRSA and vancomycin-resistant *enterococcus faecium* [13]. In the current study, we demonstrated that the bactericidal effect of HBD-3 or HBD-3 combined with ultrasound-targeted microbubble destruction, and theirs role in inhibiting the formation of antibiotic-resistant *Staphylococcus* biofilms [14], [15]. To date, murine homologs of HBDs called mouse β-defensins were isolated and characterized and more than ten different mouse β-defensins have been isolated [16]. A BLAST search of the mouse protein database with the amino acid sequence of HBD-3 revealed the highest identity to mouse β-defensin-14 (MBD-14). Futhermore, recombinant MBD-14 has also exhibited similar broad-spectrum, nanomolar microbicidal activity against various microorganisms, including gram-positive, gram- negative bacteria, the *yeast* and *candida albicans* as well as HBD-3. These data suggest that MBD-14 is the structural and functional ortholog of HBD-3 [17], [18]. Therefore, it is very useful to study the expression and regulation of MBD-14 in vivo based on its homology to the human orthologue HBD-3. Our previous studies have identified the relevant signaling pathway p38 mitogen-activated protein kinase (p38 MAPK) that contributed to the obvious release of MBD-14 in mouse osteoblasts upon contact with MRSA supernatant, the bacterial exoproducts released by *S. aureus* mainly included a large amount of toxins, which provided a theoretical basis as a promising therapeutic target in the treatment of intramedullary infection with MRSA in vivo [19].

Anisomycin produced from *Streptomyces griseolus* and *Streptomyces roseochro- mogenes* was identified in the year 1956 [20]. It was originally identified as an antibiotic against certain protozoa and fungi, which led to proposed clinical uses as a topical anticandidal and antiamoebic drug in humans [21], [22]. It has prominently been applied by a great many research groups in memory research since the 1960s, proving that it is intimately implicated in temporary amnesia for reinstated memories, learning and memory [23], [24], [25]. Independent of these ability, anisomycin has also been widely used as an activator for p38 MAPK in studies investigating the p38 MAPK signaling pathway in mammalian cells [26]. However, it remains ambiguous whether anisomycin-induced activation of the p38 MAPK signal transduction pathway has an impact on *S. aureus*-induced bone infection. Therefore, the main objective of this present study was to evaluate the efficacy of different doses of anisomycin in the treatment of osteomyelitis in a mouse model by increasing the release of MBD-14. During the experiment, bacterial contamination was followed by intramedullary injection of MRSA and development of infection was assessed by blood analyses, microbiological and histological investigations in different groups. Meanwhile, it is well-known that when bone tissue gets infected, it upsets the normal process of bone remodelling that is orchestrated by osteoclasts and osteoblasts. Recent studies demonstrated that when *S. aureus* binds to osteoblast cells, in addition to inducing apoptosis, it stunts osteoblast cell growth [27]. Amongst the most widely studied of these related signal pathways is that involving the mitogen-activated protein kinase (MAPK) family, which is known to regulate the proliferation and differentiation of osteoblasts [28]. In addition, there is convincing evidence that the activation of the MAPK family can enhance osteoblast differentiation and proliferation and, thereby, increase bone formation [29], [30]. Therefore, in the current report we also demonstrate whether anisomycin can regulate the expression of key markers of osteoblast growth and division such as alkaline phosphatase (ALP), osteocalcin (OCN) and collagen type I (COLL1) by influencing p38 MAPK signaling. In addition, we sought to identify and characterize HBD-3 released by human interfacial membranes in aseptic loosening and periprosthetic joint infection to investigate potential differences of inducible antimicrobial peptides in the presence of infection and question whether HBD-3 play an important role in the inflammatory process.

## Materials and Methods

### Bacteria and Preparation of Inocula

Infection was induced by inoculation of MRSA (ATCC 43300) which was purchased in a freeze-dried form from the American Type Culture Collection (Rockefeller, MD, USA). Species identification and susceptibility testing were performed with the Vitek 2 automated system (Bio Mérieux, Marcy I’Étoile, France). The phenotypic classification of the ATCC 43300 was further confirmed by methicillin-resistance determinant A (*MecA*) expression, which encodes PBP2a (penicillin-binding protein 2a) and mediates methicillin resistance [31]. The production of bacteria was performed as described by Lucke et al [32]. From an overnight culture of ATCC 43300 in 9 ml BBL Trypticase Soy Broth (TSB; BD Biosciences, Franklin Lakes, NJ), portions of 100 µl were transferred into sterile tubes containing 3 ml of TSB. These tubes were then incubated for 3 h at 37°C in order to obtain log-phase growth. After incubation, tubes were centrifuged for 10 min at 3000 rpm, the supernatant was decanted, and the remaining pellet was washed three with phosphate-buffered saline (PBS) and added to PBS until a McFarland standard of 6 was obtained. Colony forming units (CFU) per milliliter were confirmed by the spread plate method [14].

### Mouse Osteomyelitis Model and Operative Procedure

The use of mice protocol was approved by the Committee on the Ethics of Animal Experiments of the Shanghai Sixth People's Hospital, Shanghai Jiao Tong University School of Medicine (Permit Number: 20110128). A model of osteomyelitis in mice was established to evaluate anti-infectious effect of anisomycin in vivo. Previous pilot studies showed that acute osteomyelitis of the mouse tibia could reliably be induced with standardized concentrations of ATCC 43300 (10^3^ CFU) inoculated into the medullary cavity [32]. BALB/c, inbred, male mice 8–12-weeks old were used in all septic osteitis experiments [33]. They were kept in the animal facility of Shanghai Sixth People’s Hospital under standard conditions. The intraosseous injections were performed according to the technique performed on anesthetized mice [32], [33]. Before the operation, 60 mice had been randomly divided into 4 groups. Surgery was performed under general anesthesia by weight-adopted intraperitoneal injection of 5% pentobarbital sodium (100 mg/kg body wt), and all efforts were made to minimize suffering. Animals were prepared for surgery as follows: the two hind leg was shaved, depilated and disinfected with alcohol. To provide sterile conditions during surgery animals were placed on sterile drapes and bodies were covered with sterile sheets. Skin and fascia at the proximal tibial metaphysis were incised over 5 mm in length. With a hand-driven titanium burr, a 1-mm hole was drilled through cortical and cancellous bone in order to access the medullary cavity at the proximal metaphysis. The surrounding periosteum remained intact. The medullary cavity was bluntly reamed with a steel Kirschner wire (1.0 mm diameter). After removal, PBS containing 10^3^ CFU/10 µl of ATCC 43300 was injected with a 50-µl micro syringe (Hamilton, IL, USA) for contamination of the medullary cavity. Soft tissues were irrigated with betadine solution and skin and fascia were sutured. Animals in the control group were injected with the same volume of PBS. Subsequently, the mice were continued to be injected intraperitoneally by different doses of anisomycin for 7 days (1 time per day), and mice were injected with 0.9% NaCl solution as a control. Mice were sacrificed as described by Ren et al [34]. On day 1, day 4 and day 7 after the injection, the mice were killed and one part of infected murine tibia was removed, and fixed in 4% paraformaldehyde for hematoxylin-eosin (HE) stain and immunohistochemistry. Another part was frozen and subsequently crushed in an achate mortar under liquid nitrogen to prepare for RNA extraction, microbiological evaluation and enzyme linked immuno sorbent assay (ELISA). The following groups were investigated: Group I 0.9% NaCl+PBS/10 µl; Group II 0.9% NaCl+ATCC 43300 10^3^ CFU/10 µl; Group III 15.0 mg/kg anisomycin+ATCC 43300 10^3^ CFU/10 µl; Group IV 5.0 mg/kg anisomycin+ATCC 43300 10^3^ CFU/10 µl [20].

### Body Weight and Body Temperature

The tympanal temperature of average five animals in four groups was measured randomly using a digital thermometer daily, and body weight was determined randomly with a electronic precision scale. Animals were inspected for clinical signs of infection (appearance of soft tissue at entry site of injection, loss of mobility and joint effusion) [32].

### Blood Analyses and Microbiological Evaluation

The average five animals in four different groups were sacrificed randomly on day 1, day 4 and day 7 after the injection. Under peritoneal anesthesia, blood samples (2.0 ml) were taken from the retro orbital sinus, analyzed for routine laboratory parameters (hemoglobin and white blood cell count), and collected for culture on day 7 [35]. Bacteria isolated from swabs, blood cultures and agar plates were identified by a fully automized laboratory system for identification and resistance determination of bacteria (Vitek, Bio Mérieux, Marcy L’Etoile, France). Tibiae of the two hind legs were dissected under sterile conditions using separate instruments for preparation of the skin and the underneath tissue. All soft tissues were removed, and bones were weighed using a electronic precision scale. Randomly one half of the left tibia of every group was chilled with liquid nitrogen, crushed into fragments, and pulverized in a sterile bone mill; 50 mg of bone powder was agitated in 1.0 ml of sterile PBS for 2 min by vortex. The suspension was centrifuged for 10 sec (10,000 rpm) and 100 µl of the supernatant was withdrawn for serial (10-fold) dilutions. Agar plates were incubated at 37°C. After 24 h, CFU on agar plates were counted. The samples were analyzed for determination of *S. aureus* CFU/g bone [32].

### ELISA

100 mg fresh weight from the other half of the left hind tibia and human tissues were crushed in an achate mortar under liquid nitrogen and homogenized in 150 mM NaCl, 20 mM Tris HCl buffer, 1% Triton X-100, pH 7.5. A soluble fraction was obtained by centrifugation and 200 µl aliquots of this homogenate were examined by sandwich ELISA according to the standard protocols of the ELISA kit (EIAab Science Co., Ltd., Wuhan, China). Standards or samples were then added to the microtiter platewells (96-well) with a biotin-conjugated polyclonal antibody preparation specific for MBD-14 or HBD-3 and incubated for 2 h at 37°C. Subsequently, plates were washed again three times with PBS +0.1% Tween 20 and filled with 100 µl of avidin conjugated to Horseradish Peroxidase (HRP) in each well and incubated for 1 h at 37°C. Then a 90 µl 3.3′, 5.5′ tetramethylbenzidine, which served as the developmental agent, was added to each well and incubated within 15 minutes in the dark at 37°C followed by 50 µl of a sulphuric acid solution. ELISA was performed on each sample in triplicate. The absorbance was measured with a Synergy HT multidetection microplate reader (BioTek Instruments) at a wavelength of 450 nm. Human recombinant HBD-3 or MBD-14 served as the standard at the following concentrations: 0, 0.3125, 0.625, 1.25, 2.5, 5, 10, 20, 40, 80, 120 ng/l [19].

### Hematoxylin and Eosin Stain

One half of the right tibia of every group was fixed, dehydrated, and paraffin embedded. Paraffin sections (4 µm) were cut, mounted, and stained with HE. The slides were permanently bonded with coverslips after staining, and the digital photomicrographs were obtained. Different cells were identified based on nuclear morphology. All slices were observed separately by four independent observers in a blinded manner [36].

### Interfacial or Synovial Membrane Preparation

The tissue samples of interfacial membranes were obtained from the tissues around the femoral implants in 10 patients who had undergone revision of total hip replacement because of periprosthetic joint infection. The patients had clinical, biochemical, or operative findings that indicated bacterial infections of the artificial hip joints. All samples from 10 patients suffering from periprosthetic joint infection showed positive microbiological cultures for *staphylococcus*, including 7 males and 3 females, with a mean age of 63.0±9.8 years (range, 43–78 years). Among them, 5 cases were *S. aureus* and the other 5 cases were *S. epidermids*. The tissue samples of interfacial membranes were obtained from the tissues around the aseptically loosened femoral implants in 10 patients who had undergone revision of total hip replacement because of aseptic loosening of implants. None of the patients had clinical, biochemical, or operative findings that would have indicated bacterial infections of the artificial hip joints. None of the patients had any major technical problems, such as femoral misalignment [36]. There were four women and six men, with a mean age of 64.3±5.7 years (range, 55–73 years). The control synovial samples of the hip joints were obtained from 10 patients who underwent primary hip arthroplasty because of the fresh fracture of the femoral neck, including 3 males and 7 females, with a mean age of 73.9±8.7 years (range, 61–86 years). No patient had clinical, biochemical, or operative findings that would have indicated bacterial infections, osteoarthritis, rheumatoid arthritis, or other arthritis of the hip joints. Following dissection, one half of each tissue biopsy was immediately frozen in liquid nitrogen and the other half was fixed in 4% formalin, dehydrated in graded concentrations of ethanol, and embedded in paraffin. Subsequently, all samples were examined for the production and expression of HBD-3 [37]. The study was approved by the ethical committee of the Shanghai Sixth People’s Hospital, Shanghai Jiao Tong University School of Medicine, and the study was performed according to the standards on human experimentation in accordance with the Helsinki Declaration of 1975 as revised in 1983 with written informed consent of the participants.

### Immunohistochemistry

Immunohistochemistry for detection of the expression of MBD-14 in a septic osteitis mouse model and distribution of HBD-3 in synovial or interfacial membrane was performed. After five animals in each groups were sacrificed randomly on day 1, day 4 and day 7, the other half of the right hind tibia was removed and decalcified with EDTA. Human specimens were dehydrated and embedded in paraffin. All deparaffinized sections were blocked with 3% hydrogen peroxide for 10 minutes at room temperature to inhibit endogenous peroxidases. Immunohistochemical staining was performed using polyclonal primary antibodies against MBD-14 (1∶500, EIAab Science, Wuhan, China) or HBD-3 (1∶500, Novus Biologicals, Littleton, CO, USA). This was followed by incubation with biotinylated secondary antibodies and a peroxidase-labeled streptavidin-biotin staining technique (DAKO, Glostrup, Denmark). Afterwards the respective antibody against the antimicrobial peptide, reveals a brown reaction product. Sections of mouse skin served as positive controls [33], [38]. Negative controls were carried out by absorption of the primary antibody with recombinant protein (100 µg/ml). For HBD-3, normal human skin was used as an additional positive control [37].

### Real-time Polymerase Chain Reaction (PCR)

For real-time PCR, frozen human samples or the other half of left hind tibiae in mice were crushed in an achate mortar under liquid nitrogen, and total RNA was isolated using an RNeasy Mini kit (Qiagen, Valencia, CA, USA) according to the manufacturer’s instructions with an additional DNase I (Fermentas)-treatment step to eliminate residual genomic DNA. Total RNA (1 µg) was reverse transcribed using a Fermentas RevertAid First Strand cDNA Synthesis kit (Thermo Fisher Scientific, Inc., Waltham, MA, USA). Real-time PCR was performed on an ABI 7500 Fast machine (Applied Biosystems, Courtaboeuf, France) using Roche FastStart Universal SYBR-Green Master (Rox) (Roche Applied Science, Indianapolis, IN, USA). The reactions were performed using cDNA templates and specific forward and reverse primers (Table 1). The specificity of the amplification reaction was determined by analyzing the melting curves. The relative amount of target gene expression was normalized to β-actin as an internal control. The quantification of gene expression was based on the cycle threshold value of each sample, which was calculated as the average of 3 replicate measurements for each sample analyzed as previously described [14], [39].

**Table 1:** Primers nucleotide sequences used in this study.

Target gene	Forward (5′ to 3′)	Reverse (5′ to 3′)
HBD-3	GGAGCTCTGCCTTACCATT	ACGCTGAGACTGGATGAA
ALP	CCAACTCTTTTGTGCCAGAGA	GGCTACATTGGTGTTGAGCTTT
OCN	CTGACCTCACAGATCCCAAGC	TGGTCTGATAGCTCGTCACAAG
COLL1	TGTGCCAATTTCATCAAGGTCC	CTCTTCCCACGACCGTTTTCA
β-actin	AATGGGTCAGAAGGACTCCT	ACGGTTGGCCTTAGGGTTCAG

F, forward. R, reverse.

### Western Blot Analysis

Proteins extracted from human specimens were extracted using the Total protein Extraction kit according to the manufacturer’s instructions (ProMab Biotechnologies, Inc., Richmond, CA, USA). Proteins were quantified using the BCA protein assay kit (Pierce Biotechnology, Inc., Rockford, IL, USA). Samples containing equal amounts of protein (40 µg/lane) were run on 12% polyacrylamide gels, transferred onto nitrocellulose membranes (Whatman, Dassel, Germany) and blocked for 1 h with 5% non-fat milk in TBST (Tris-buffered solution containing 0.1% Tween-20) at room temperature. The membranes were then soaked with primary antibodies (HBD-3, 1∶300, Novus Biologicals, NB200-117) overnight at 4°C followed by secondary antibody (Goat Anti Rabbit IgG/HRP, 1∶5000) incubation for 1 h at room temperature. Finally, the membranes were reacted with enhanced chemiluminescence reagents and band densities were determined using Gel Pro 4.0 analyzer software (Media Cybernetics Inc., Bethesda, MD, USA) and the integrated optical density (IOD) was calculated as previously described [19].

### Statistical Analysis

Data were expressed as the means ± standard deviation (SD) derived from at least 3 independent experiments. Statistical analysis between groups were performed by one-way ANOVA. The level of *p*<0.05 was considered to indicate a statistically significant difference. SPSS software (version 19.0; IBM, Chicago, IL, USA) was used for statistical analysis [15].

## Results

### Body Weight, Body Temperature and Blood Analyses

Body weight slightly decreased in all groups in this experiment. Body temperature remained stable in all groups during the experimental period and showed no significant differences among the different groups (Table 2). The counts of WBC reached the highest values on day 4 after surgery. At this time, the counts of WBC of group II were the highest in all the groups. Moreover, the lowest numbers of WBC were observed in the group I, followed by the group III and group IV. However, no significant difference was observed between the group III and group IV. During this week following operation, the level of hemoglobin decreased in all groups. However, no significant difference was found at the same time (Table 3).

**Table 2:** Mean values of body weight and temperature of different groups.

	Body weight/g				Temperature/°C			
	Day 0	Day 1	Day 4	Day 7	Day 0	Day 1	Day 4	Day 7
Group I	18.9±0.3	18.8±0.5	18.7±0.5	18.4±0.4	37.0±0.4	37.2±0.3	37.2±0.4	37.0±0.3
Group II	18.9±0.3	18.8±0.6	18.6±0.5	18.3±0.6	37.0±0.4	37.3±0.4	37.4±0.3	37.2±0.6
Group III	18.9±0.3	18.8±0.4	18.6±0.2	18.5±0.4	37.0±0.4	37.1±0.4	37.0±0.3	37.2±0.3
Group IV	18.9±0.3	18.9±0.3	18.9±0.5	18.5±0.5	37.0±0.4	37.2±0.4	37.1±0.5	37.3±0.5

The random five animals were sacrificed on day 0 as control and no differences can be observed between the different groups.

**Table 3:** Mean values of hemoglobin and white blood cell count of different groups.

	Hemoglobin g/dl				White blood cells/nl			
	Day 0	Day 1	Day 4	Day 7	Day 0	Day 1	Day 4	Day 7
Group I	14.7±0.5	14.5±0.5	14.0±0.3	14.3±0.9	5.5±1.5	6.9±1.1	7.2±2.0*	6.4±1.6*
Group II	14.7±0.5	14.5±0.6	14.1±0.5	14.2±0.6	5.5±1.5	7.9±1.1	10.8±2.1	10.5±2.4
Group III	14.7±0.5	14.6±0.4	13.8±1.0	14.1±1.2	5.5±1.5	7.2±2.0	7.8±1.8*	7.5±1.2*
Group IV	14.7±0.5	14.4±0.4	13.9±0.7	14.4±0.9	5.5±1.5	7.4±1.3	8.1±1.5*	7.8±1.1*

* Significant to Group II at the same time point, *p*<0.05. The random five animals were sacrificed on day 0 as control.

### Microbiological Evaluation

#### Blood cultures and microbiological smears

None of the blood cultures obtained at sacrifice tested positive on ATCC 43300 in any group on day 7. Smears taken from the entry site of injection at the proximal tibia of groups I were found to be negative. In contrast, 100% of the smears of group II and group IV and 75% of group III were positive on ATCC 43300 on the seventh day postoperatively (Table 4). Bacteria isolated from swabs and agar plates were identified to be ATCC 43300.

**Table 4:** Microbiological results of the left tibiae determined on day 7 of sacrifice.

CFU/g		Group I	Group II	Group III	Group IV
Culture ofblood	Positive	0	0	0	0
Cultures ofsmears	Positive	0	5*	4*	5*

* Tested positive on ATCC43300.

#### CFU/g bone

**Figure 1:**
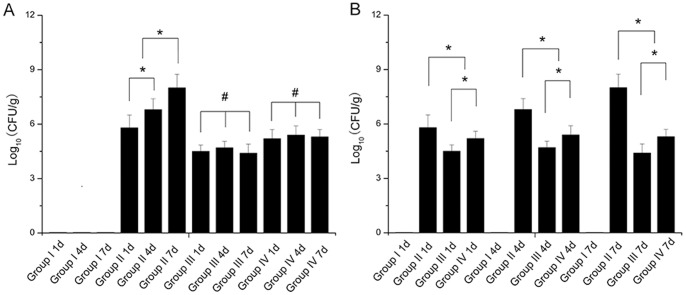
Amount of CFU per gram of bone, quantified in pulverized bone from left tibia postoperation. The mice were not contaminated with MRSA and received PBS served as controls (group I). The medullary cavities of tibiae in mice were contaminated with MRSA and different doses of anisomycin (15.0 mg/kg, group III and 5.0 mg/kg, group IV) were followed. The animals that received phosphate- buffered saline (PBS) were served as group II. (A) Same treatment conditions for different periods of time; (B) different treatment conditions for the same periods of time. All bones of group I and one of group III were sterile. Values are the mean ± standard deviation (SD). * Statistically significant differences between the 2 groups, *p*<0.05; # no significant difference, *p*>0.05.

All the tested group II showed massive bacterial growth of bone cultures (agar plates) after the operation. Mean CFU counted on agar plates in group II was far above 10^7^ CFU on the seventh day postoperatively, significantly higher than any other days after operation in group II. No bacteria was cultured from pulverized bones in group I. One sample of group III remained completely sterile after 24 h of incubation on the seventh day postoperatively, and the others of group III revealed bacterial colony formation. The average amount of CFU determined per gram infected bone of group III and IV was found to be significantly lower than that of group II at different timepoints postoperatively. Meanwhile, the average count of CFU of group III was significantly lower compared to group IV observed in different timepoints. As shown in Fig. 1B, the difference between group III and group IV was significant on the first day postoperatively, and the same findings were observed at other times. Nevertheless, results of all tested cultures of group III (or group IV) have no statistical significance in the growth of ATCC 43300 between two groups at the different timepoints after surgery (Fig. 1A).

### Histologic Evaluation

**Figure 2:**
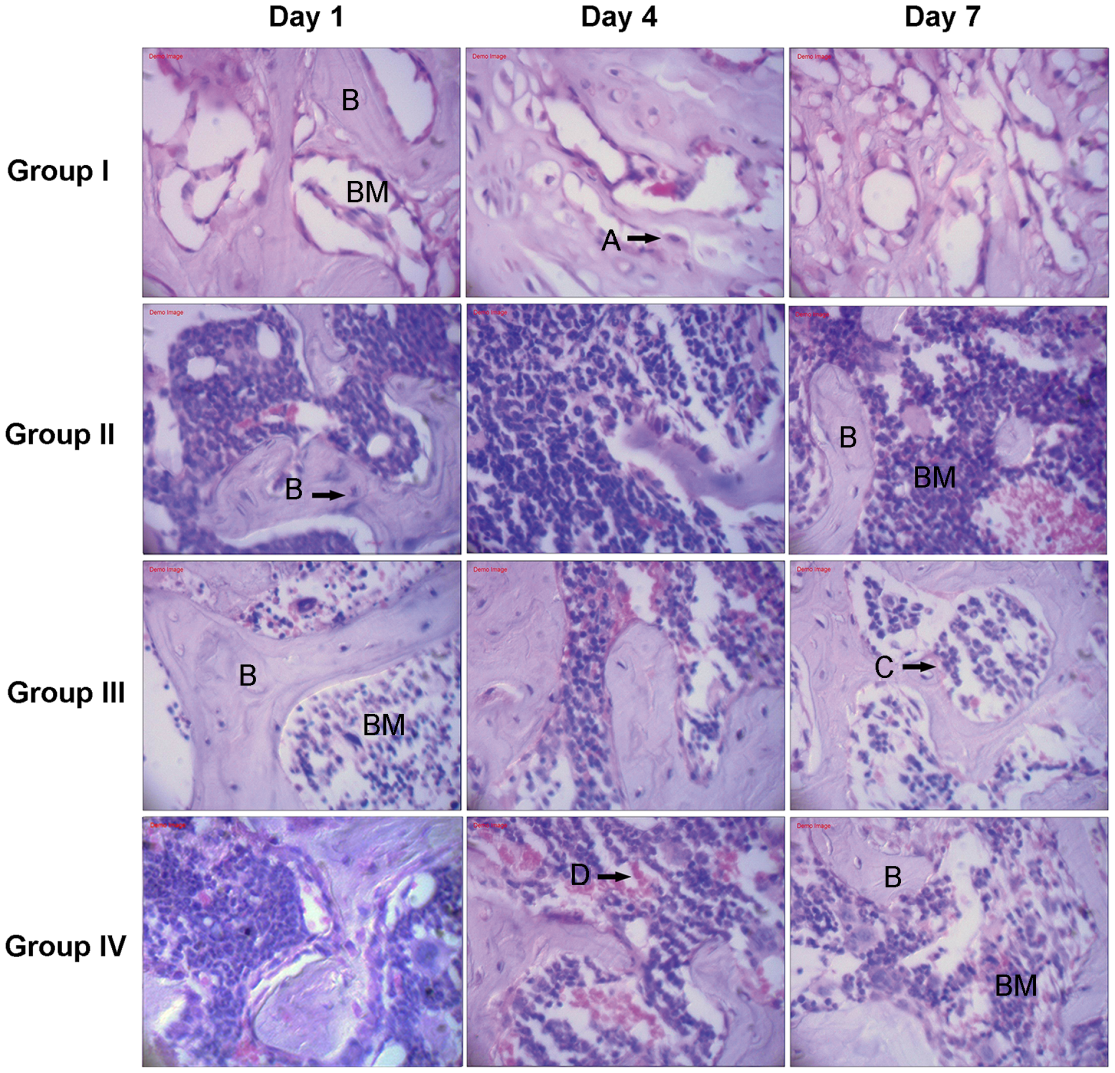
Tissues were stained with hematoxylin and eosin to visualize tissue architecture in different groups. *S. aureus* induced an increased number of neutrophils in inflammatory reaction, while systemic application of p38 agonists anisomycin reduced these responses [B: Bone; BM: Bone marrow]. Osteoblasts (A), osteocytes (B), neutrophils (C) and red blood cells (D) were observed in the tissues. Original magnification×400.

As depicted in Fig. 2, the histologic evaluation revealed that morphologically, at least four types of cells were observed in the infected mouse tibiae, including osteocytes, osteoblasts, neutrophils and red blood cells. All HE staining of group II showed typical signs of bone infection with the most immediate inflammatory response, including the recruitment of additional immune cells, such as neutronphils at the site of infection after the operation. HE slices of group I displayed no detectable signs of bone infection. In contrast to group II, histomorphological appearances of infection in group III or IV were found to be significantly reduced at the proximal metaphysis of the tibiae at different timepoints after surgery, especially in the group III.

### Immunohistochemistry

**Figure 3:**
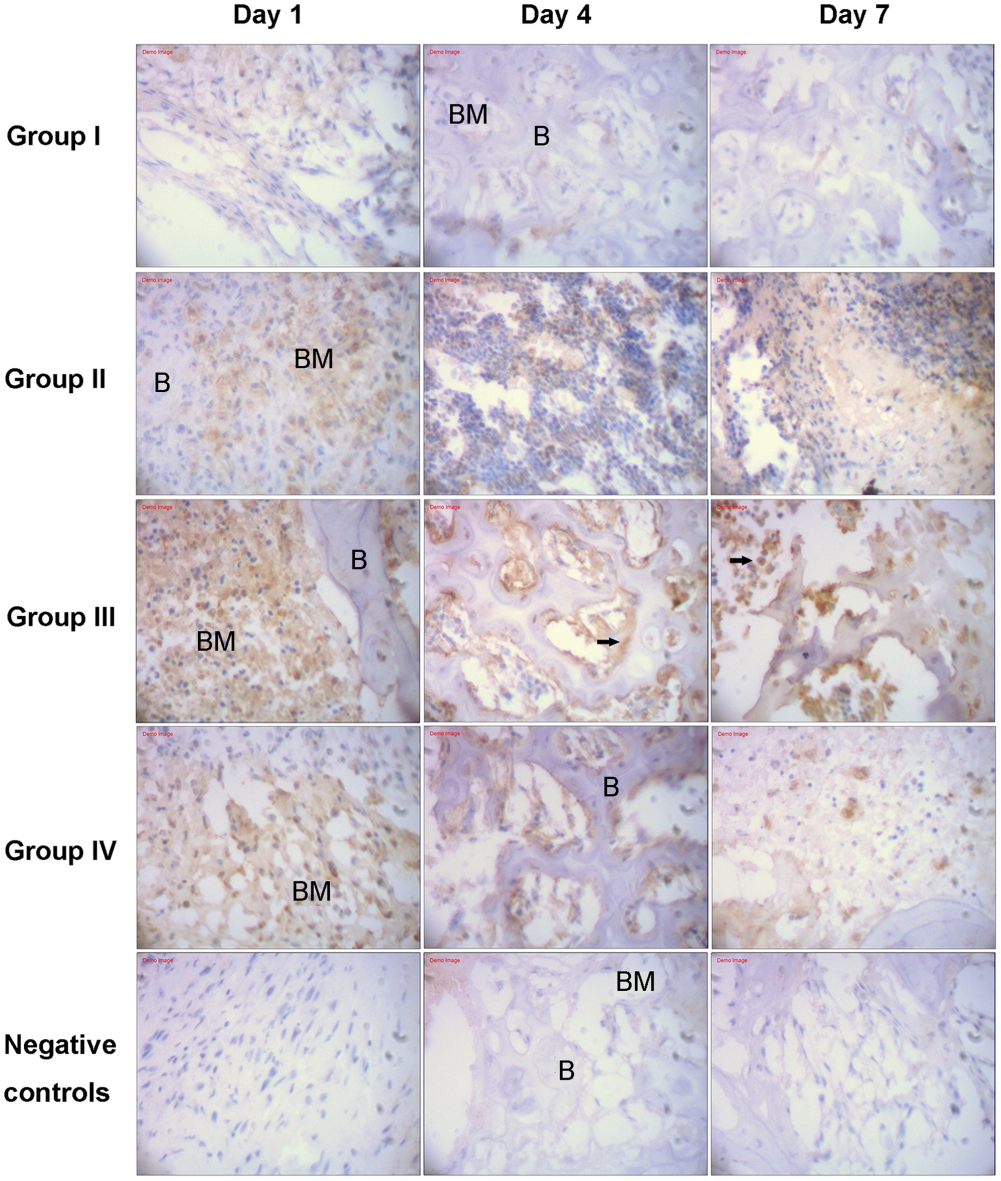
Immunhistochemical analysis of Mouse β-defensin-14 (MBD-14) expression in different groups after introsseous contamination with *S. aureus*. ATCC 43300 stimulated increased immunohistochemical-positive cells, while systemic application of p38 MAPK agonists anisomycin subsequently resulted in further improvement of the expression of MBD-14 in the pericellular matrix of endosteal osteoblasts and osteocytes (black arrows) [B: Bone; BM: Bone marrow]. Original magnification×400.

**Figure 4:**
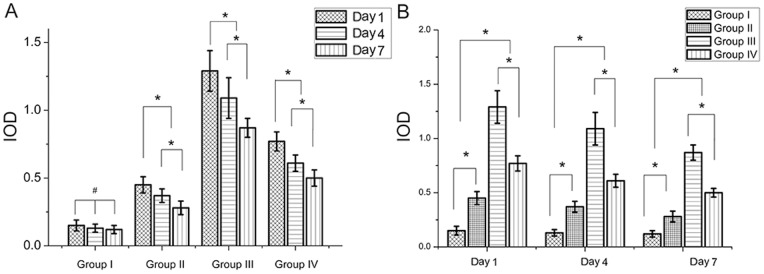
Quantitative integrated optical density (IOD) analysis of the expression of MBD-14 by immunohistochemical staining. (A) Same treatment conditions for different periods of time after *S. aureus* contamination; (B) different treatment conditions for the same periods of time. Data are the means ± SD. * Statistically significant differences between the 2 groups, *p*<0.05; # no significant difference, *p*>0.05.

As displayed in Fig. 3, immunohistochemistry in different groups visualized a clear staining of MBD-14 in different bone marrow cells and confirmed systemic application of p38 MAPK agonists anisomycin increased the expression of MBD-14 protein on day 1, day 3 and day 7 after ATCC 43300 contamination. In group I, cross sections of murine proximal tibiae showed a weak immunoreactivity to MBD-14 antibody. The application of *S. aureus* into the medullary canal of murine tibiae resulted in an increased expression of MBD-14 in two main locations. Firstly, immunoreactivity was presented in bands in the mineralized bone matrix itself at the border between bone and bone marrow. In each case, the highest expression was detected just below the endosteal cell line. Secondly, immunoreactivity to MBD-14 was detected within the cytoplasm of osteocytes and osteoblasts present in each of the histological sections. There was intensive staining in the bone, indicating a general inflammatory response to the injected bacteria. As displayed in Fig. 4A, in group II, the expression of MBD-14 in day 1 was the highest at all time points following *S. aureus* stimulation, and the same findings were observed when different doses of anisomycin were used in group III or IV. Furthermore, an obvious increase expression of MBD-14 was observed after systemic application of different doses of anisomycin compared with only intraosseous injection of *S. aureus* (group II), but the higher dose group (group III) showed the higher expression of MBD-14 in different time points (Fig. 4B).

### ELISA

**Figure 5:**
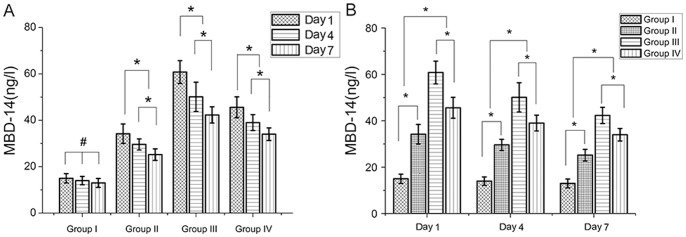
ELISA was performed to assess the amount of MBD-14 released in different groups after exposure to ATCC 43300. (A) Same treatment conditions for different periods of time; (B) different treatment conditions for the same periods of time. Data are the means ± SD. * Statistically significant differences between the 2 groups, *p*<0.05; # no significant difference, *p*>0.05.

As shown in Fig. 5, compared to the control group, bacterial contamination increased the release of MBD-14 by two and a half times on day 1, two times on day 3 and day 7 respectively, suggesting an effective contamination of the medullary cavity of these bones. Results also showed that anisomycin stimulation in the higher dose rapidly increased the expression of MBD-14 level up to the highest of 60.8±4.9 ng/l 24 h postoperatively, indicating the fast and effective expression, and the level of MBD-14 on day 7 was the lowest at all time points. Anisomycin stimulation in low dose group has the same tendency of changes with high dose group. However, a significant difference was observed between two groups at the same periods of time.

### Osteoblast-associated Genes

**Figure 6:**
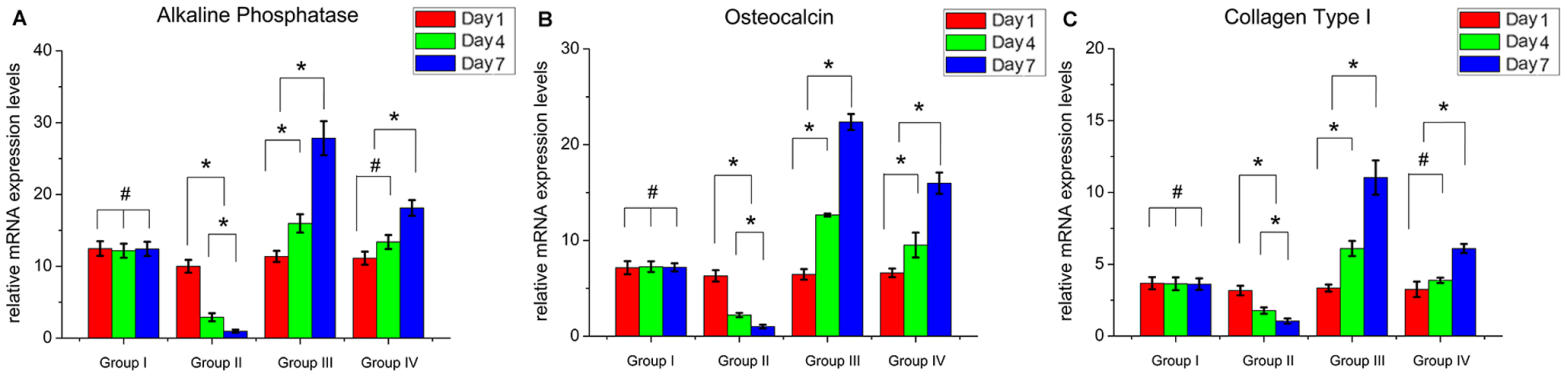
Real-time PCR analysis of the expression of the early osteogenic markers alkaline phosphatase (A), osteopontin (B) and collagen type I (C). The expression levels of three osteogenic markers normalized to the internal standard gene β-actin. The mRNA levels shown are the relative mRNA levels compared to the mRNA levels in the lowest level sample. Data are the means ± SD. * Statistically significant differences between the 2 groups, *p*<0.05.

As depicted in Fig. 6, analysis of osteogenesis was carried out by quantitative real time PCR and the target gene expression in different groups was relative quantification and calculated as a fold change compared with the lowest level sample [40]. Tibiae of the infected mice showed a significant decrease in the expression of osteoblast-associated genes in group II on day 1, day 3 and day 7, including ALP, OCN and COLL1, compared with the other three groups. When mice were treated with only intraosseous injection of *S. aureus* (group II) for different periods of time, the levels of three osteoblast-associated genes positively correlated with the duration. The expression of ALP mRNA in group III slightly decreased on day 1, remarkably induced on day 4 and the highest level was maintained and peaked with more than 2-fold stimulation at day 7 compared with group I, and the same findings were observed in the expression of OCN and COLL1 mRNA. Meanwhile, the levels of mRNA for ALP, OCN and COLL1 were significantly higher in group III than group IV throughout the 4 or 7 days of treatment.

### Increased Release of HBD-3 in Infected Interfacial Membrane after *Staphylococcus* Contamination

**Figure 7:**
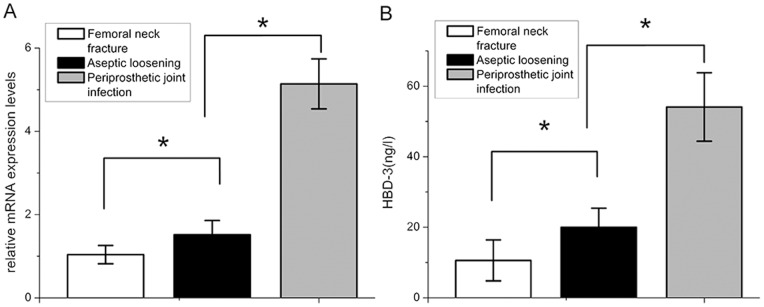
Real-time PCR analysis of the expression levels of Human β-defensin-3 (HBD-3) mRNA (a). The expression levels of HBD-3 normalized to the internal standard gene β-actin. The mRNA levels shown are the relative mRNA levels compared to the mRNA levels in the control group. **ELISA experiments** (b) demonstrated the basal HBD-3 expression levels in samples of the synovial membrane. In the case of bacterial bone infection, increased levels of HBD-3 protein were determined, suggesting a bacterial influence on the induction of HBD-3. Data are the means ± SD. * Statistically significant differences between the 2 groups, *p*<0.05.

**Figure 8:**
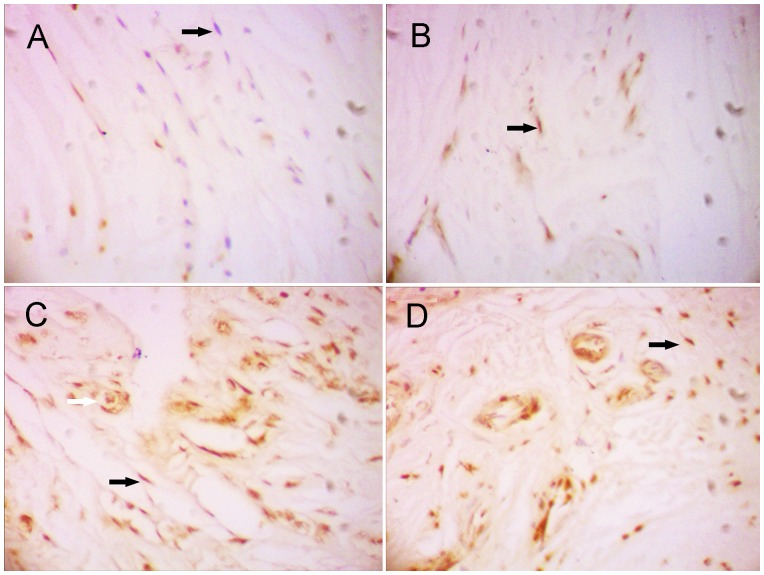
Human β-defensin-3 (HBD-3) was expressed and induced in bacteria- infected interfacial membrane by immunohistochemistry analysis. Samples from femoral neck fracture (A), aseptic loosening (B) and periprosthetic joint infection (C, *S. aureus* and D, *S. epidermids*) were treated and revealed HBD-3 distribution in all examined tissue samples. Immunohistochemical examinations revealed a strong immunoreactivity to HBD-3 antibody in tissue samples of infected interfacial membranes. Staining was primarily found or detected in the extracellular matrix of fibroblasts (black arrows) and osteocytes (white arrow). Original magnification×400.

**Figure 9:**
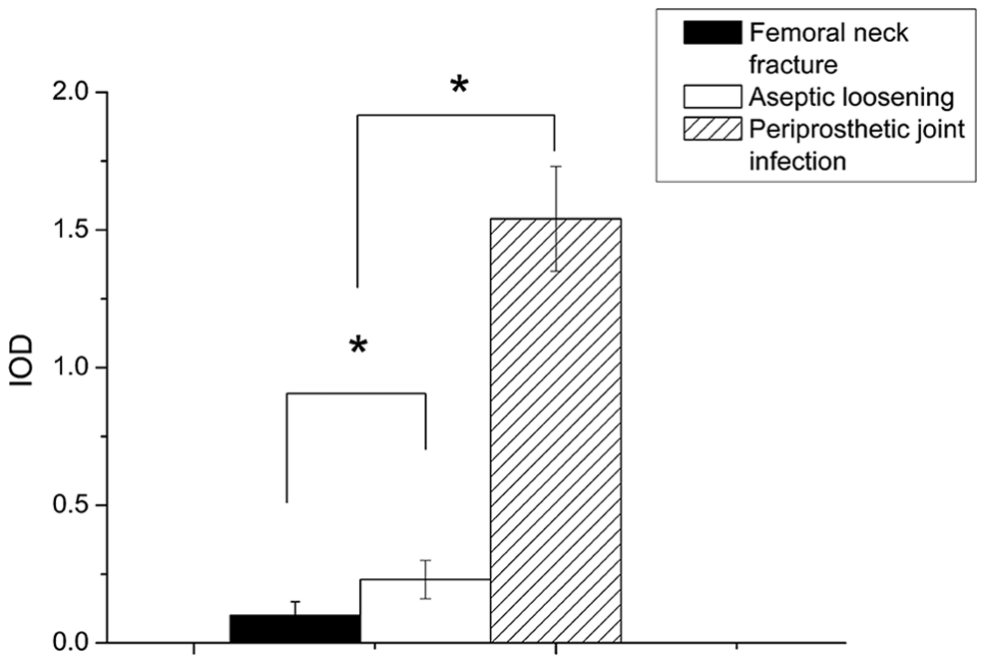
Quantitative integrated optical density (IOD) analysis of the level of HBD-3 by immunohistochemical staining. Samples from femoral neck fracture, aseptic loosening and periprosthetic joint infection were assessed. Values are the mean ± SD, * Statistically significant differences between the 2 groups, *p*<0.05.

**Figure 10:**
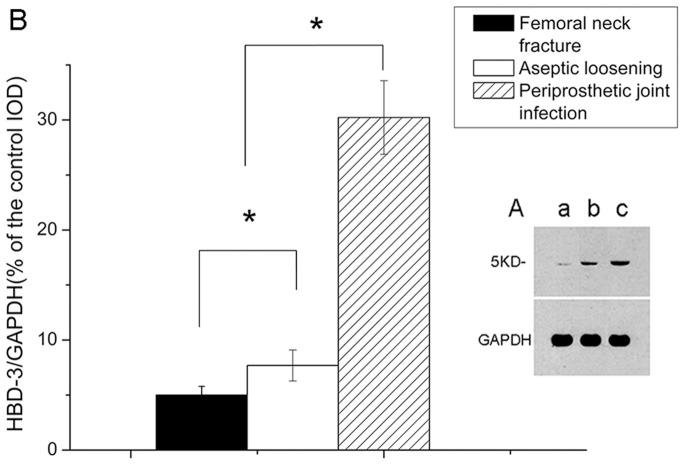
Western blot analysis assays verified HBD-3 proteins expression elevated in human infected interfacial membranes. Shown is a representative blot of HBD-3 (A). Samples from femoral neck fracture (a), aseptic loosening (b) and periprosthetic joint infection (c) were treated for the indicated time. Pre-incubation with specific antibody revealed only a weak detection in the fracture (a) and aseptic loosening (b) group. Quantitative IOD analysis of HBD-3 protein levels by western blot analysis (B). The densities of protein levels in different groups were normalized to those of the GAPDH groups. Values are the mean ± SD, * Statistically significant differences between the 2 groups, *p*<0.05.

By real time-PCR, the expression of HBD-3 mRNA were lower in the non- infectious interfacial membrane in aseptic loosening and the synovial membrane after femoral neck fracture than the interfacial membrane in periprosthetic joint infection (Fig. 7A). After bacterial challenge, HBD-3 release was increased approximately fivefold in infectious tissues compared with the synovial membranes, and more than 2 times compared with the non-infectious interfacial membranes (Fig. 7B). Immuno- histochemistry revealed the staining intensity of HBD-3 localized in fibroblasts and osteocytes in inflamed interfacial membranes significantly higher than not-inflamed interfacial membranes (Fig. 8, 9). Moreover, no obvious difference was observed between different disease-causing bacteria, *S. aureus* and *S. epidermids*. In addition, all synovial membranes show, sometimes, HBD-3 immunoreactivity that was only present in fibroblasts in the superficial layers of synovial membranes (Fig. 8). As shown in Figure 10, the analysis of the HBD-3 concentration showed single bands in the relevant regions for the antibacterial peptide investigated. In case of bacterial colonization with *staphylococcus*, an significant increase in HBD-3 protein level compared with non-inflamed tissues was found. Moreover, there was significant difference in the amount of HBD-3 between the interfacial membrane in aseptic loosening and the control synovial membrane in femoral neck fracture.

## Discussion

The aim of this study was to investigate a new idea that serves as a systematic antimicrobial peptides delivery system to prevent bone infections. Therefore, in this study, the efficacy of anisomycin to increase the release of MBD-14 and inhibit bacteria growth was evaluated in a mouse osteomyelitis model. In experiments, mouse medullary cavity was contaminated with ATCC 43300 which is characterized by their high affinity to bone, their rapid induction of osteonecrosis, and resorption of bone matrix [41]. Subsequently, acute destructive osteomyelitis developed in mice that received an intramedullary inoculum of 10^3^ CFU of the pathogen and simultaneously no signs of osteomyelitis were seen in animals that received sterile saline. The onset of infection could also be ascertained after sacrifice by microbiological investigations and histological analysis. Microbiological cultures obtained from group II after sacrifice showed the massive growth of ATCC 43300 detached from the infectious tibiae. It may be concluded that the chosen mouse model is suitable to reliably induce experimental osteomyelitis. From group III and IV, previously inoculated bacterial growth of positive cultures performed on nutrient agar was obviously killed or significantly reduced in these animals, and onset of infection was effectively prevented. In these animals from group III and IV, development of infection was not completely hindered. However, the extent and intensity of infection were significantly reduced as shown by histological results. The results show that anisomycin, the p38 MAPK activator, obviously increased the release of MBD-14 and suppressed *S. aureus* growth in this mouse model of osteomyelitis and a higher concentration achieved a stronger inhibitive effect.

We next investigated the effect anisomycin has on new bone formation with bacterial contamination. Bone formation is typically characterised by the sequential expression of a series of bone formation markers including ALP, OCN and COLL1. Early studies demonstrated ALP is an enzyme found in osteoblasts and is a biochemical indicator of early stage osteogenesis. Moreover, during the process of osteogenesis, osteoblasts secrete proteins that form a matrix for newly proliferated osteoblasts to adhere to [27]. Such matrix proteins include OCN and COLL1. To do this RNA was isolated from uninfected and *S. aureus*-infected groups (from group I to group IV) following 1, 4 and 7 days. In the current study we found that *S. aureus* inhibited osteogenesis by inhibiting osteoblast expression of ALP, OCN and COLL1 over a 7 day period in group II. However, a high dose of anisomycin in group III, even low-does in group IV, significantly recovered osteoblast expression of ALP, OCN and COLL1, suggesting that anisomycin, the p38 MAPK activator, not only has the good antibacterial property but also does good to osteogenesis.

Simultaneously, this study is the first one to investigate the expression and production of HBD-3, the known human inducible defensins, in the inflamed interfacial membrane of periprosthetic joint infection. Previous study showed that HBD-3 can exert their antimicrobial activity by interacting with membranes of metabolically active bacteria, or by electrostatic charge-based mechanisms of membrane permeation [42], [43], [44]. Recent studies found that inducible HBD-3 can be produced in human fibroblasts and osteocytes after contact with pathogenic bacteria, such as *S. aureus* [12], [45]. In this study, HBD-3 was expressed and up-regulated by contact with pathogenic bacteria, *S. aureus* or *S. epidermids*, and showed high levels of message for the peptide in all samples of inflamed interfacial membrane in the presence of periprosthetic joint infection using immunohistochemistry, real time PCR, ELISA and western blot analysis. In comparison with infection sites, our work revealed that the expression of HBD-3 is lower in all the interfacial membranes from prosthetic aseptic loosening. Moreover, we were able to detect the peptide only in partial synovial membranes analysed by immunohistochemistry, compared with infected interfacial membranes, perhaps because its concentration is below the limit of detection of our antibody. These findings are in line with recent studys showing that infections can reduce or turn off the expression of HBD-3, suggesting that its production depends on the status of local bacterial microflora [12], [19], [45]. The results obtained in this paper are valuable for the fact that *staphylococcus* contributes over two-thirds of the identified organisms in periprosthetic joint infection. The inducibility of HBD-3 makes it as a stronger candidate for antimicrobial defence in the periprosthetic joint infection.

In the previous studies, Röhrl [18], [46] et al confirmed that MBD-14 played a dual role during immune response. For one thing MBD-14 contributed to the local innate immune response by combating microbial invasions; for another previous studies revealed the capacity of MBD-14 had ability to chemoattract a broad spectrum of leukocytes, such as monocytes, macrophages, neutrophils, immature dendritic cells and T cells in a CCR6- and CCR2- dependent manner [18], [46]. Moreover, it was shown that HBD-3, the structural and functional ortholog of MBD-14, activates antigen presenting cells (APCs) via TLR1 and TLR2 in a NF-κB-dependent manner [47]. So the MBD-14 contributes to the innate and adaptive immune response in the role as chemoattractants. In addition to their chemotactic and antimicrobial activity, there is evidence suggesting that β-defensins participate in the regulation of host innate and adaptive immune responses. Presicce [48] et al provided data showing the capacity of hBD-1 to induce the expression of maturation markers (e.g., MHC class II and CD83), costimulatory markers (e.g., CD80, CD86, and CD40), and pro- inflammatory cytokines (e.g., TNF, IL-6, and IL-12p70) in human monocyte-derived dendritic cells. Thus, we hypothesize that MBD-14 produced by mouse osteoblasts could promote chemotactic recruitment and activation of a broad spectrum of leukocytes and APCs to sites of infection and inflammation, and regulate host innate and adaptive immune responses. Subsequently, it is necessary to study the mechanism based on which MBD-14 regulates the host innate and adaptive immune responses against microbial infection in the osteomyelitis mouse model and further studies are required to confirm these hypotheses.

Several studies have suggested that anisomycin-induced activation of a p38 MAPK signaling pathway possibly influences lymphocyte activation, proliferation, cell cycle and others signaling pathways, such as c-Jun NH2-terminal kinase (JNK) or extra cellular signal-regulated kinase1/2 (ERK1/2) [20], [49], [50]. Other recent research findings have shown that anisomycin inhibits the behaviors of T cells and the transplantation rejection in a model of mouse allogeneic skin transplantation [20]. Whether these functions have relations with the inhibition of bacterial infection should be verified by further studies. In addition, when p38 MAPK is activated throughout the organism, possible side effects will certainly occur as p38 MAPK signal pathway are so frequently occurring in almost all body cells, tissues and organs. However, the main purpose for the primary study only showed that p38 MAPK may play a critical role in a murine osteomyelitis model and p38 MAPK may provide a potential therapeutic target. Further studies are required to solve this problem.

In the current report we demonstrate that anisomycin significantly induced the expression of key markers of osteoblast growth and division such as ALP, OCN and COLL1. One reason for this might be the well-established antibacterial activities of anisomycin, which could reduce damage to the osteoblasts during *S. aureus* bone infection. Another possible mechanism could be that anisomycin exerts its influence on osteogenesisis, including osteoblast proliferation and differentiation, by modulating the p38 MAPK pathway. In the future, further experiments and studies are required to test these hypotheses.

One shortcoming of the animal experiment is the relatively small sample size. However, our experiments have been conducted rigorously with appropriate controls, replication, and relevant datas were collected and abstracted in our research at least from 3 independent experiments. Moreover, the problem will be avioded in the further study. The other shortcoming of this study is using the synovial membrane samples from hip joints of fresh femoral neck fracture as the control groups [51]. The most ideal control is the interfacial membranes or synovial membranes from patients who have had a previous arthroplasty without periprosthetic joint infection or aseptic loosening, and an alternative control is a sample of normal synovial membrane. However, the controls are not easily obtained from patients who have had a previous arthroplasty without aseptic loosening or periprosthetic joint infection because revision total hip replacement is rarely performed on these patients after arthroplasty. In addition, there are rare chances to get normal synovial membranes from normal hip joints. Another alternative control is the synovial membranes from hip joints of fresh femoral neck fracture, although traumatic synovial membrane shows slight in flammation and slightly more proinflammatory cytokines than normal synovial membrane [52], [53]. More importantly, compared with normal synovial membrane, the status of the traumatic synovial membrane would probably decrease, rather than increase, the difference between interfacial membrane and control synovial membrane. A recent study found that serum of multiply injured patients has higher antibacterial capacity than that of healthy donors [54]. Thus, the difference between the interfacial membranes and control synovial membranes may be larger if normal synovial membrane has been available as a control. Meanwhile, the present study is limited by the number of samples studied. However, the results clearly show in all cases investigated that the inflamed interfacial membrane from periprosthetic joint infection induced by *staphylococcus* leads to induction and expression of HBD-3. It may be hypothesized that purified or recombinant HBD-3 may be an ideal agent in the therapy of *staphylococcus*-induced infection when applied via injection at the site of infection.

Inflammatory diseases, both periprosthetic joint infection and non-infectious aseptic loosening, are a prominent problem in clinical revision arthroplasty. Accurate and early detection of infectious and non-infectious is of great importance for elucidation of the cause of the disease, early prevention of the onset of complications, and rapid implementation of a tailored therapeutic regimen [55], [56]. In the study, the significant difference of the expression of HBD-3 which has been connected with antibacterial tasks has also been found between inflamed interfacial membranes and not inflamed interfacial membranes. In the future, a feasible application of HBD-3 may even help doctors make a distinction between periprosthetic joint infection and aseptic loosening by intraoperative histological biopsy of the interfacial membranes. Another more practical approach, in further clinical studies, maybe to radiolabel HBD-3 with the goal of assessing the potential of HBD-3 radiolabeled as a radiopharmaceutical for imaging periprosthetic joint infection because the anti- microbial peptide HBD-3 selectively bind to the bacterial cell membrane [55], [56].

In summary, our findings may provide a feasible and effective method of antibacterial treatment for mouse osteomyelitis through systemic application of p38 MAPK pathways agonists anisomycin and may have important clinical significance. In addition, the expression of HBD-3 in periprosthetic joint infection was significantly higher than aseptic loosening under inflammatory conditions, so we believe that HBD-3 represents an interesting antibacterial agent for treatment and diagnosis of orthopedic nosocomial infections.
